# Commentary: Predictors of professional help-seeking intention toward depression among community-dwelling populations: a structural equation modeling analysis

**DOI:** 10.3389/fpsyt.2024.1363160

**Published:** 2024-03-15

**Authors:** Kenneth Po-Lun Fung, Soyeon Kim

**Affiliations:** ^1^Department of Psychiatry, University of Toronto, Toronto, ON, Canada; ^2^Toronto Western Hospital, University Health Network, Toronto, ON, Canada; ^3^Department of Psychiatry and Behavioural Neurosciences, McMaster University, Hamilton, ON, Canada; ^4^Waypoint Research Institute, Waypoint Centre for Mental Health Care, Penetanguishene, ON, Canada

**Keywords:** stigma, depression, culture, Asian, Chinese

## Introduction

Depression is one of the most common mental disorders, estimated to have resulted in around 280 million DALYs globally in, 2019 (1). Timely, appropriate intervention for depression is important to decrease morbidity and mortality from suicide. One approach to understanding help-seeking behaviours is to examine a person’s intention to get help, which is influenced by a person’s underlying attitudes, perceived subjective norms, and perceived behavioral control according to the Theory of Planned Behavior (2). As mental health help-seeking behaviors are generally low, especially in developing countries, the paper investigated contributing factors influencing Professional Help-Seeking Intention (PHSI) for depression among, 2000 people in Wuhan City, China. The study found that positive professional help-seeking attitudes and depression knowledge positively correlated with PHSI, while stigma negatively correlated with PHSI. Using structural equation modelling, direct and indirect impacts of various factors on PHSI were also examined, including family functioning and severity of depression. The study contributes towards building evidence around the complexity of factors affecting help-seeking intentions, especially alluding to the important interactive roles of stigma, culture, and different levels of consideration from the individual to the family. The findings and study limitations point toward critical gaps in the literature, which will be the focus of this commentary.

## Cultural considerations of stigmatizing narratives

The study findings are consistent with the literature suggesting that stigma is one of the key barriers to people seeking help through the shaping of underlying attitudes towards mental illness (3), including negative perceptions about its causes, such as personal weakness, and its treatment, such as acceptability of seeing mental health professionals, taking medications, and/or undergoing psychotherapy. As the authors indicate in the discussion, this can be understood as reflecting differences in a person’s underlying *explanatory model of illness*, which varies across cultures with nuances. This can be more directly probed using instruments like the Explanatory Model Interview Catalogue (EMIC) and EMIC Stigma Scale, which also encompasses social aspects of stigma (4, 5). This medical anthropological approach applied in cultural psychiatry was first championed by Kleinman in studying depression in China. While general stigmatizing attitudes may be common across cultures, as the authors opined, Chinese-specific cultural beliefs and values, including Confucianism, may impact stigma, such as beliefs about maintaining “face” and social harmony, accepting fate and destiny, etc. Exploring the role of culture embedded in mental health concepts and associated behaviours through empirical studies such as this article is critical to designing anti-stigma interventions that can work more effectively in various cultural contexts.

## Internalized stigma

Internalized stigma involves presuming others’ disparaging attitudes towards mental illness, believing in these assumptions, and applying them to oneself. Its impact can be best elucidated through the use of specific internalized stigma scales as well as qualitative methods. This is especially relevant as depression is an illness marked by negative self-perceptions. Furthermore, culture impacts on how one construes the psychological “self,” such that the “interdependent self,” a “self” defined in relation to others, becomes salient in collectivistic cultures, alongside the autonomous, independent self as usually conceived in Western cultures. This opens a new vantage point to investigate the impact of internalized stigma, which needs to consider the effect of stigma on one’s interdependent self, as well as for those related to the depressed person, i.e., “stigma by association” or “affiliate stigma” experienced by family members (6). Family may serve as an important mediating factor of stigma in collectivistic cultures, such as East Asian culture, as mental illness may be perceived as a family flaw, inducing shame for the whole family (7–9).

## Stigma and the family

One of the study’s strengths is the inclusion of family functioning in the modeling. As the authors indicate, in East Asian culture, grounded in Confucianism, filial piety, hierarchy in the family, and family-oriented interdependence are important considerations. Accordingly, developing and using more culturally appropriate methods for assessing family functioning and the internalized stigma of family members is critical. For instance, the Family APGAR index used in the study emphasizes elements more consistent with Western values, such as the direct expression of affection, which is more nuanced in cultures that utilize high-context and indirect communications.

## The intersectional and social lens

Further, it is important to consider the impact of gendered roles and expectations within the family along with other social attributes, such as families situated in urban versus rural settings, where family reputation may become even more paramount for the latter. Informed by intersectionality, social identities such as race, ethnicity, culture, gender, sexuality, education, and SES can have a complex synergistic negative impact on stigma and PHSI, leading to further MH disparity (10, 11). For example, the impacts of stigma may be moderated by gendered roles (12–14), whereby in East-Asian cultures, women may be more expected and socially permitted to express their mental health difficulties or seek help. In a larger social context, attitudes toward seeking help depend on the person believing that socially and culturally competent and appropriate services are available (15).

## Discussion

With modernization and globalization, progress has been made in decreasing the stigma against mental illness. Yet, it is evident that stigma remains a significant issue in many parts of the world and for many communities within developed countries. A knowledge-based approach through increasing mental health literacy, while helpful, is quite limited and, indeed, may not change stigmatizing attitudes unless cultural issues are addressed. As cultural issues are context-dependent, they must be addressed at the individual, family, and community levels, considering other social and systemic factors. This entails researchers identifying Western cultural assumptions about the perception of the self, family, and societal norms and developing research methodologies that are culturally appropriate and encompass mixed quantitative and qualitative methods.

Moving forward, we should adopt a balanced and flexible approach that considers the unique cultural perspectives of individual, family, and societal dynamics. This approach should remain committed to fostering greater compassion and acceptance surrounding mental health and its treatment, guided by Equity, Diversity, and Inclusion (EDI) principles and cultural competence. Furthermore, it is imperative to continue expanding our understanding of mental health and illness by conducting rigorous scientific research on treatments such as acupuncture, Tai-chi, or Traditional Chinese Medicine (TCM) in combination with Western approaches, especially for the Asian population, the focus of the paper. Additionally, we can draw from mindfulness and acceptance interventions rooted in Asian philosophy, which may prove effective in treating depression as well as reducing both internalized and social stigma (13, 14, 16–18). These interventions can help build individual and community resilience, which is only beginning to be explored.

## Author contributions

KF: Writing – review & editing, Writing – original draft, Conceptualization. SK: Writing – review & editing, Writing – original draft, Conceptualization.
